# Untargeted Metabolomic Profiling of Colonic Mucosa in Individuals with Irritable Bowel Syndrome

**DOI:** 10.3390/biomedicines13030629

**Published:** 2025-03-05

**Authors:** Patrycja Krynicka, Mariusz Kaczmarczyk, Karolina Skonieczna-Żydecka, Daniel Styburski, Konrad Podsiadło, Danuta Cembrowska-Lech, Krzysztof Dąbkowski, Anna Deskur, Wiesława Rogoza-Mateja, Małgorzata Ławniczak, Andrzej Białek, Anastasios Koulaouzidis, Wojciech Marlicz

**Affiliations:** 1 Department of Gastroenterology, Pomeranian Medical University, 71-252 Szczecin, Poland; krynickapatrycja@gmail.com (P.K.); wiesiarogoza@op.pl (W.R.-M.); bialekab@gmail.com (A.B.); wojciech.marlicz@sanprobi.pl (W.M.); 2 Department of Biochemical Science, Pomeranian Medical University, 71-460 Szczecin, Polandkarolina.skonieczna.zydecka@pum.edu.pl (K.S.-Ż.); 3 Sanprobi sp. z o.o. sp.k., 70-525 Szczecin, Polandkonrad.podsiadlo@sanprobi.pl (K.P.); danuta.cembrowska@sanprobi.pl (D.C.-L.); 4 Department of Clinical Research, University of Southern Denmark (SDU), 5230 Odense, Denmark; 5 Research Unit, Department of Surgery, Odense University Hospital (OUH), 5000 Svendborg, Denmark

**Keywords:** irritable bowel syndrome, metabolomics, mucosal microbiota, disorders of gut–brain axis, DGBI, biomarkers, liquid chromatography–mass spectrometry

## Abstract

**Background:** Irritable Bowel Syndrome (IBS) is a complex disorder characterized by altered gut–brain interactions, with gastrointestinal microbiota and metabolic dysregulation playing key roles in its pathophysiology. Identifying specific metabolic alterations within the colonic mucosa may enhance our understanding of IBS and contribute to improved diagnostic and therapeutic approaches. **Methods:** This cross-sectional study analyzed the metabolomic profiles of colonic mucosal biopsies from 44 IBS patients assessed with ROME IV criteria and 69 healthy controls undergoing colonoscopy. Untargeted metabolomic profiling was conducted using liquid chromatography–mass spectrometry (LC-MS), and differential metabolite analysis was performed via fold-change calculations and machine learning-based classification. **Results:** IBS patients exhibited distinct mucosal metabolic profiles, with significantly elevated levels of N-acetylneuraminic acid and 1-palmitoylglycerol, suggesting compromised epithelial integrity and increased gut permeability. In contrast, cis-4-hydroxycyclohexanecarboxylic acid, a metabolite associated with protective mucosal functions, was reduced. Random Forest analysis identified these metabolites as key discriminatory features between IBS and control groups, reinforcing their potential role as biomarkers for IBS-related mucosal alterations. **Conclusions:** Our study highlights the unique metabolomic signatures of IBS at the mucosal level, emphasizing the role of microbial metabolites in disease pathology. These findings may facilitate the development of novel diagnostic tools and targeted therapeutic strategies, advancing personalized management for IBS patients.

## 1. Introduction

Irritable Bowel Syndrome (IBS) is a disorder of the gut–brain interaction (DGBI) characterized by symptoms such as abdominal pain, bloating, and altered bowel habits without identifiable organic causes [1]. As a chronic condition, IBS considerably impacts the quality of life for those affected and imposes a significant economic and psychological burden due to its high prevalence and requirement for long-term management [2,3]. The Rome IV criteria define IBS as a disorder characterized by recurrent abdominal pain associated with changes in stool form or frequency, with symptoms present for at least six months and active in the last three months. Subtypes include IBS-C (constipation-predominant), IBS-D (diarrhea-predominant), and IBS-M (mixed). These criteria are widely used for IBS classification in clinical and research settings. Epidemiological studies estimate that IBS affects approximately 4–21% of the global population, though prevalence varies widely between countries and demographic groups [4,5,6,7].

The pathophysiology of IBS is multifactorial and remains incompletely understood, posing challenges to developing effective treatments [8]. Recent research highlights the critical role of microbial and metabolic alterations among various contributing factors, which have been linked to the hallmark of IBS symptoms, such as increased gut permeability, low-grade inflammation, and abnormal gastrointestinal motility [9,10]. The rising prevalence of post-infectious IBS in the era of COVID-19, often accompanied by overlapping conditions such as functional dyspepsia, highlights the growing need for comprehensive management strategies targeting the gut–brain axis [11,12].

The metabolome—representing the complete set of small molecules produced by both the host and its microbiota—has emerged as a valuable lens through which these interactions can be explored. Metabolomic profiling provides a unique opportunity to identify biomarkers of health-related conditions and better elucidate the mechanisms behind pathological states [13,14]. However, the data concerning the metabolome profiles at the level of colonic mucosa in patients with IBS are scarce.

In the current study, we focused on individuals presenting for endoscopic evaluations and aimed to uncover metabolic alterations specific to IBS. We screened patients for IBS diagnosis using the Rome IV criteria, enabling the identification of distinct metabolic profiles within the colonic mucosa biopsies. Our findings could pave the way for novel diagnostic and therapeutic strategies, ultimately advancing personalized management approaches for IBS patients.

## 2. Patients and Methods

### 2.1. Study Design

The study was designed as a cross-sectional observational analysis focusing on individuals diagnosed with IBS according to the Rome IV criteria [5]. The Rome IV validated questionnaire version in the Polish language was licensed by the Rome Foundation (License Agreement, 7 February 2022). The primary objective was to characterize the metabolome of the colonic mucosa in selected individuals and to identify any distinct metabolomic pattern associated with IBS. The study aimed to ensure that the observed alterations were associated explicitly with IBS by excluding participants with any detectable organic diseases or other confounding conditions. Participants with any detectable organic gastrointestinal diseases, including inflammatory bowel disease (IBD), colorectal cancer, celiac disease, or microscopic colitis, were excluded from the study. The study was conducted in compliance with the Helsinki Declaration. The protocol was approved by the Pomeranian Medical University (PMU) ethics committee in Szczecin (resolution No. KB-0012/197/19; date 19 February 2019), and all participants gave written informed consent to participate in the present study.

We recruited patients between 2021 and 2023 from individuals presenting for gastrointestinal endoscopic examinations at the PMU Hospital nr 1 Endoscopy Unit in Szczecin, Poland. To avoid selection bias, random sampling was performed. To ensure the integrity and reliability of the data, specific exclusion criteria were established that could have potentially biased the study results. These were (i) age under 18, (ii) pregnancy, (iii) presence of severe comorbidities, (iv) history of IBD, (v) history of immunosuppressive therapy, (vi) presence of ileo- or colostomy, (vii) hospitalization at a time of enrollment, (viii) referral for advanced endoscopic procedures (e.g., polypectomy, endoscopic mucosal or sub-mucosal resection/EMR, ESD/ ix), incomplete questionnaires, or lack of informed consent. Participants who had used antibiotics, prebiotics, or probiotics in the three months prior to sample collection were excluded to minimize the influence of external factors on metabolomic profiles.

A total of 2070 individuals of the West Pomeranian (Poland) Cohort were screened, of which 436 were diagnosed with IBS based on the Rome IV criteria as previously reported [5]. Among them, 44 individuals fulfilling the Rome IV criteria for IBS and undergoing colonoscopy were randomly selected, of whom mucosal biopsies of the large intestine were obtained for metabolomic analysis. A control group of 69 healthy individuals undergoing colonoscopy and colonic biopsies, without DGBI or organic gastrointestinal disorders, was also included for comparative analysis. Control participants underwent colonoscopy for preventive screening or due to non-specific gastrointestinal symptoms that were ultimately not linked to organic pathology. Overall, one hundred thirteen (n = 113) participants provided colonic mucosal biopsies for the study.

### 2.2. Sample Collection

One hundred thirteen (n = 113) participants provided colonic mucosal biopsies for the study, including 44 individuals diagnosed with IBS and 69 participants from a control group. These samples were retrieved during colonoscopy procedures conducted by experienced gastroenterologists at the Endoscopy Unit of Clinical Hospital No. 1 in Szczecin, Poland. Before the colonoscopy, patients underwent preparation following established, standard medical protocols, which included cleansing the bowel using a polyethylene glycol (PEG) solution. Biopsy samples were strategically collected from various colon segments using biopsy forceps, ensuring that areas with noticeable inflammatory alterations were avoided in the control group. Each participant provided 2 to 3 mucosal tissue fragments, roughly 2 to 3 mm in size, which were then securely placed into sterile Eppendorf tubes filled with DNA Shield (Zymo Research, Irvine, CA, USA), enabling the storage of the samples at 4 °C until analysis.

### 2.3. Liquid Chromatography–Mass Spectrometry Analysis

A mixture of methanol, water, and acetonitrile (100 μL) in 50:25:25 *v*/*v*/*v* proportions with deuterated internal standards was added to biopsy samples. The sample was shaken at 2000 rpm at 4 °C for 30 min to dissolve the metabolites in the solution. Subsequently, samples were centrifuged for 4 min at a speed of 4000 rpm and a temperature of 4 °C. The supernatant was transferred to the chromatography vial and, as such, analyzed on the same day by liquid chromatography–mass spectrometry. QC samples were prepared by mixing test samples in equal proportions and prepared in the same way as the test samples. The LCMS analysis was conducted on an ExionLC liquid chromatograph equipped with a binary pump, autosampler, and column thermostat coupled with a Triple TOF 6600+ mass spectrometer (Sciex, Framingham, MA, USA). The separation was carried out on a Phenomenex Luna^®^ Omega 1.6 μm polar C18 150 × 2.1 mm column for 45 min in gradient separation. The mobile phases were Phase A—water with 10mM ammonium acetate—and Phase B—acetonitrile with 0.1% formic acid. The column injection was 2 μL, and the column temperature was 20 °C. The phase flow was 0.2 mL/min. Spectral analysis was performed in the positive ion mode with a capillary voltage of 5500 V, Curtain gas (CUR) at 25 psi, Ion source gas 1 (GS1) at 45 psi, Ion source gas 2 (GS2) at 60 psi, and the ion source temperature at 400 °C. The mode negative ions had a capillary voltage of 4500 V, Curtain gas (CUR) at 25 psi, Ion source gas 1 (GS1) at 45 psi, Ion source gas 2 (GS2) at 60 psi, and the ion source temperature at 350 °C. The spectrometer collected spectral data in SWATH mode.

The spectra were analyzed using SCIEX OS software (version: 2.0.0.45330) and integrated SCIEX All-In-One HR-MS/MS, NIST, and our own databases. The identification of compounds was based on the analysis of retention time, high resolution *m*/*z*, and analysis of fragments formed as a result of metabolite degradation in a collision cell. The resulting datasets were preprocessed to ensure quality control, including normalization, log transformation, and scaling, as implemented in MetaboAnalystR (version 4.0.0). The metabolite abundances of IBS patients and controls and IBS-C and IBS-D subtypes were compared using fold-change (FC) analysis. FC was computed as the ratio between the mean abundance in the IBS and control groups or between the mean abundance in IBS-D and IBS-C. A threshold of log2FC of ≥1 or ≤−1 was used (corresponding to an FC threshold of ≥2 or ≤0.5). The volcano plot was used to report the results of the fold-change analysis, showing the metabolites as a function of the *p*-value (−log10 *p*-value) and the fold-change value (log2 fold change).

### 2.4. Random Forest Classification and Feature Importance Analysis

The machine learning Random Forest (RF) algorithm was applied using nested cross-validation to find the best hyperparameters and to evaluate the model while minimizing the risk of overfitting. The nested cross-validation consisted of training data (70%) and testing data (30%) created using a stratified algorithm to maintain a balanced class representation. Data were split into training and testing sets using a stratified algorithm to preserve a balanced class representation. The training data were further divided into two folds, which were used to select the best hyperparameters using a grid search approach. The following RF hyperparameters were optimized: the number of features randomly sampled for splitting, the number of decision trees, the maximum tree depth, and the minimum number of samples per leaf node. The RF models were evaluated using the area under the receiver operating characteristic curve (AUC). Feature importance was assessed by randomly permuting each feature in the test data. The AUC and a complementary 1-AUC measure were calculated for each permuted predictor.

The training data were also used to generate a dataset with randomly shuffled outcome labels (Y-scrambled data). All steps described above were repeated using Y-scrambled data, allowing us to compare the predictive potential of models trained on real and scrambled data, thereby excluding the possibility of capturing noise rather than real patterns. The entire process was repeated 100 times to calculate bootstrap (1000 samples), 95% confidence intervals (CIs) of evaluation, and feature importance metrics. R statistical package was used for all analyses (version 4.4.0 [15]). RF models were built using the Ranger R package (version 0.16.0).

## 3. Results

**Figure 1:**
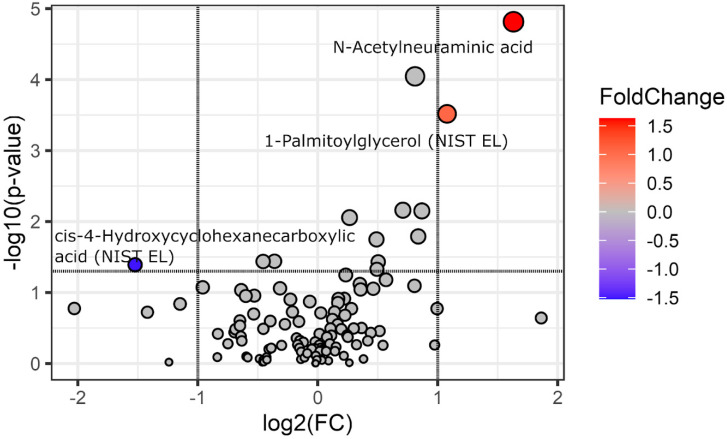
Volcano plot of fold-change and statistical significance for differential features between IBS and control groups. Positive values of the log2FC indicate a higher metabolite level in IBS patients; negative values indicate a higher metabolite level in non-IBS individuals; *p* values, reported as −log10, were obtained from a *t*-test. Points are colored according to fold-change, while point size reflects the −log10 (*p*-value). Only metabolites exceeding the fold-change threshold (log2 fold change ≥ 1 or ≤−1, indicated by vertical lines) and *p*-values < 0.05 (corresponding to −log10 of 1.30—a horizontal line) are highlighted.

Except for age (IBS: 52 ± 16 years, non-IBS 59 ± 13 years, *p* = 0.029), there were no significant differences between the groups. The demographic characteristics of enrolled patients are presented in Table 1. Using the fold-change analysis, we first compared the metabolite levels of IBS and controls. N-acetylneuraminic acid (log2 fold change [log2FC] = 1.63, *p* = 0.00003, false discovery rate [FDR] Q = 0.003) and 1-Palmitoylglycerol (log2FC = 1.08, *p* = 0.0002, Q = 0.009) were among the metabolites with higher concentrations in the IBS group (Figure 1). In contrast, the control group had higher levels of cis-4-Hydroxycyclohexanecarboxylic acid (NIST EL) (log2FC = −1.52, *p* = 0.023, Q = 0.279). Although it was below the log2FC threshold of 1, corresponding to a fold-change of 2, glycine was also more abundant in the IBS group and statistically significant (*p* = 0.00006, Q = 0.003). The full-fold change analysis results are shown in Supplementary Table S1.

**Figure 2:**
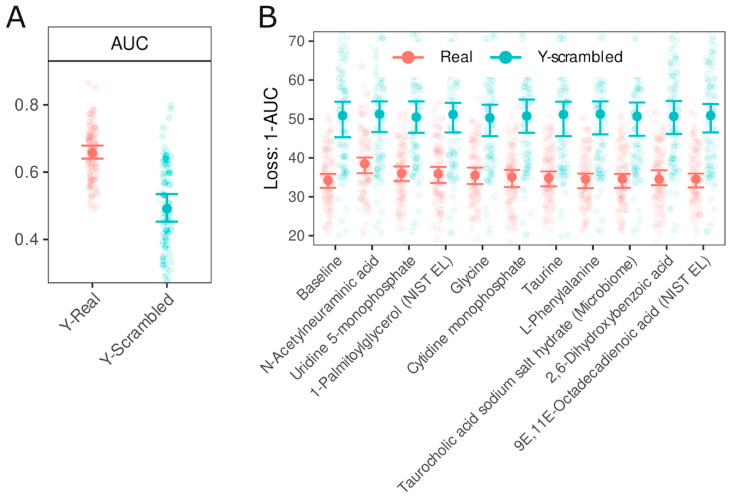
Evaluation of Random Forest model performance and feature importance based on AUC and 1-AUC metrics. Panel (**A**): Test data AUC ROC values with bootstrap 95% CI for models trained on real data (Y-real) and Y-scrambled data from 100 iterations. Panel (**B**): Feature importance analysis—10 most influential predictors, ranked by a median 1-AUC value. Features were permuted in the test set, and the corresponding reduction in AUC was calculated for models trained on Y-real (red) and Y-scrambled (blue) outcome data. Error bars represent bootstrap 95% CI. The baseline refers to AUC values obtained without permutation (100 iterations themselves). Comparing the 95% CIs of permuted features against the baseline enables statistical assessment of feature importance.

Next, we used the Random Forest machine learning algorithm to identify key metabolites that distinguished the IBS patients from non-IBS individuals. The median area under the curve (AUC) of the ROC for the real data (Y-Real) and scrambled data (Y-scrambled) was 0.66 (95% CI 0.64–0.68) and 0.49 (95% CI 0.45–0.54), respectively, Figure 2A. Feature importance analysis revealed that N-acetylneuraminic acid was the most important predictor, and its 95% CI of 1-AUC did not overlap with the 95% CI of the baseline. The second most crucial metabolite was uridine-5-monophosphate, followed by 1-palmitoylglycerol. The feature importance values (1-AUC) obtained from the model trained on scrambled data centered around 50%, without any noticeable pattern.

The fold-change analysis was also conducted on a group of IBS patients to identify metabolites that differ between the two IBS subtypes, IBS-C and IBS-D. The classification of IBS into subtypes such as IBS-C and IBS-D is based on Rome IV criteria. These subtypes have been widely recognized in IBS research and clinical practice and were not introduced as a novel categorization in this study. Although some metabolites exceeded the fold-change threshold, for example, eleutheroside E + NH3 (log2FC = 2.15, *p* = 0.489, Q = 0.921), glycodeoxycholic (log2FC = −3.39, *p* = 0.682, Q = 0.921), and sodium glycochenodeoxycholate (log2FC = −2.31, *p* = 0.730, Q = 0.921), the difference in abundance between IBS-C and IBS-D were not statistically significant. The full results of the fold-change analysis comparing IBS-C and IBS-D are presented in Supplementary Table S2.

## 4. Discussion

Colonic mucosal biopsies provide detailed insights into pathological processes, effectively identifying changes in intestinal barrier function, inflammation, and metabolism [16]. A standard fold-change analysis and a Random Forest machine learning classification analysis, using a stringent and controlled approach, including nested cross-validation and scrambled data, pinpointed essential metabolites that set IBS patients apart from controls. Neu5Ac, Uridine-5-monophosphate, and 1-Palmitoylglycerol exhibited the most potent discriminatory power, a finding also evident in the fold-change analysis results. This consistency underscores the potential biological relevance of these metabolites and positions them as candidate biomarkers for IBS.

Most metabolomics studies have reported data based on fecal or plasma analyses, focusing on patients with IBD and cancer [17,18]. While these approaches provide valuable systemic insights, they may not fully capture metabolic alterations at the tissue level [19]. Colonic biopsies offer a more detailed understanding of the biochemical environment in the intestinal mucosa. This approach may help uncover disease-specific metabolic pathways and improve the identification of potential therapeutic targets [20,21]. While fecal studies help evaluate overall microbiota composition and their metabolites, such as short-chain fatty acids (SCFAs), bile acids, and amino acid derivatives, biopsies uncover more detailed information about metabolic function at the epithelial level [22]. Although microbiota composition and function were not analyzed in our current study, mucosal metabolomics provides insights into the local metabolic environment, which complements microbiome studies based on fecal or plasma samples.

Accordingly, Neu5Ac and 1-palmitoyl glycerol, found in our study to be elevated in IBS patients, could be directly related to the function of the colonic barrier and related low-grade inflammation process. Including these metabolites in future diagnostic panels could facilitate non-invasive diagnostic methods, reducing the burden of repetitive invasive procedures like colonoscopies [23,24].

Elevated Neu5Ac levels are consistent with findings by Li et al. [25], who associated its presence with chronic inflammation and increased gut permeability. Neu5Ac also affects immune responses and epithelial glycoprotein composition, potentially linking localized epithelial dysfunction to broader microbial alterations. This aligns with research by Wang et al. [26] and Liu et al. [27], which highlighted Neu5Ac’s role in sialic acid metabolism and its implications for gut microbiota composition, function, and inflammation. Similarly, elevated 1-palmitoylglycerol levels in IBS patients point to its role in promoting inflammation and compromising the intestinal barrier, as supported by studies like that of Li et al. [28] and McGuckin et al. [29]. Conversely, reduced levels of cis-4-hydroxycyclohexanecarboxylic acid, a protective microbiota byproduct, suggest impaired mucosal defence mechanisms, increasing susceptibility to epithelial damage [30].

Colonic biopsies could provide precise insights into localized metabolic changes [20,21,22,31]. Our current data provide a novel perspective on the role of mucosal microbiota in IBS and contrast with those presented by Iribarren et al. (2024) or Kirk et al., who primarily focused on characterizing the plasma and fecal metabolomes in participants with DGBI [32,33]. While both studies recognize the importance of microbial and metabolic alterations in IBS pathophysiology, our results suggest a more pronounced role of mucosal-associated metabolites in symptom manifestation and disease progression.

Fraser et al. [13] also recently emphasised systemic metabolic alterations. One key point of divergence between our current study and the Fraser et al. report is the approach to microbiota analysis [34]. Fraser et al. relied on fecal and plasma metabolome analyses, which may not fully capture the microbial metabolites present within the mucosal layer [13]. In our study, we employed a detailed assessment of the mucosal metabolome, which more directly reflects the microbial interaction with the host’s immune system and intestinal epithelium, thereby influencing local inflammation and visceral hypersensitivity at the level of GI barrier function [35]. In detail, we identified specific metabolites, including Neu5Ac, 1-Palmitoylglycerol, and cis-4-hydroxycyclohexanecarboxylic acid, which are primarily associated with localized epithelial changes and often undetectable in fecal studies. Of note and in contrast to Fraser et al.’s study, we were not able to show differences between individuals with either IBS-C and IBS-D subtypes.

Combining colon biopsy and fecal metabolomics data could enhance diagnostic accuracy and lead to more targeted treatments [31].

Since colonoscopy is not required for IBS diagnosis and is only recommended in individuals with alarm symptoms or for cancer screening and surveillance, mucosal biopsies taken during colonoscopy in individuals with IBS may serve as a source of metabolic profiling, providing reassurance for both patients and clinicians. Moreover, while not yet part of clinical guidelines, mucosal metabolomics may offer a future complementary role in therapeutic decision-making, potentially guiding more targeted treatment approaches.

Our study does have limitations. The small sample size and lack of control over long-term dietary and lifestyle factors may confound results. The cross-sectional design restricts causal conclusions, and the focus on large intestinal biopsies excludes potential findings from other gut regions. Although untargeted metabolomics provided a broad overview, specific key metabolites may have been missed due to detection limits. Additionally, the moderate performance of the Random Forest model suggests the need for integration with other datasets, such as microbiome profiles, to improve diagnostic precision.

Future studies should focus on longitudinal designs, integrating metabolomics with proteomics and transcriptomics to clarify causal relationships. Including duodenal and small intestine biopsies could expand our understanding of metabolic changes in IBS and overlapping DGBI syndromes. Our study highlights the significance of colonic biopsies in identifying localized metabolic changes in IBS and improving our understanding of IBS, opening up new avenues for diagnostic and therapeutic strategies and moving us closer to personalized management for DGBI-affected individuals.

## Figures and Tables

**Table 1:** Participants characteristics.

Characteristic	IBS N = 44 ^1^	Other N = 69 ^1^	*p* ^2^
Sex			0.175
Females	30 (68%)	38 (55%)	
Males	14 (32%)	31 (45%)	
Age (years)			0.029
Mean (SD)	52 (16)	59 (13)	
Body mass (kg)			0.741
Mean (SD)	77 (18)	77 (15)	
Height (cm)			0.538
Mean (SD)	168 (10)	168 (13)	
BMI (kg/m^2^)			0.932
Mean (SD)	27.0 (5.4)	27.7 (8.5)	
DM (Yes/No)	7 (16%)	9 (13%)	0.783
Hypertension (Yes/No)	14 (32%)	32 (46%)	0.169

^1^ N (%), ^2^ Fisher’s exact test; Wilcoxon rank sum test.

## Data Availability

The datasets used and/or analyzed during the current study are available from the corresponding author on reasonable request.

## Supplementary Materials

The following supporting information can be downloaded at: https://www.mdpi.com/article/10.3390/biomedicines13030629/s1, Table S1: Fold change analysis and t-test for comparing IBS and healthy subjects. Table S2. Fold change analysis and t-test for comparing IBS-C and IBS-D.
