# Supplementary material for: Untargeted Metabolomic Profiling of Colonic Mucosa in Individuals with Irritable Bowel Syndrome
Source: Biomedicines. 2025 Mar 5;13(3):629. doi: 10.3390/biomedicines13030629 (PMC11940239; doi:10.3390/biomedicines13030629)
Supplement: Supplementary file 1 [file biomedicines-13-00629-s001.zip › biomedicines-3488625-supplementary.pdf]

Supplementary Table S1. Fold change analysis and t-test for comparing IBS and healthy subjects.

Metabolite	Fold Change	log2(FC)	t.stat	P	-log10(p)	FD
Mannitol	0.24	-2.03	-1.48	0.14	0.85	0.5
3-Hydroxybenzoic acid	3.64	1.86	1.20	0.23	0.64	0.6
N-Acetylneuraminic acid	3.10	1.63	4.39	0.00	4.49	0.0
cis-4-Hydroxycyclohexanecarboxylic acid (NIST EL)	0.35	-1.52	-2.31	0.02	1.65	0.2
Canrenone	0.37	-1.42	-1.40	0.16	0.79	0.5
cis-5,8,11-Eicosatrienoic acid (NIST EL)	0.42	-1.24	0.06	0.95	0.02	0.9
10-Gingerol	0.45	-1.15	-1.51	0.13	0.87	0.5
1-Palmitoylglycerol (NIST EL)	2.11	1.08	3.81	0.00	3.62	0.0
Citrulline	1.99	0.99	1.34	0.18	0.73	0.5
Hyocholic Acid (Microbiome)	1.97	0.97	0.58	0.56	0.25	0.8
Isovaleryl-L-carnitine (chloride) (Microbiome)	0.51	-0.96	-1.87	0.06	1.19	0.4
Sphinganine	1.82	0.87	2.81	0.00	2.22	0.1
Ursodeoxycholic acid, MNH4	1.79	0.84	2.46	0.01	1.80	0.2
Taurocholic acid sodium salt hydrate (Microbiome)	0.56	-0.84	0.24	0.81	0.09	0.9

Linoleic acid	0.56	-0.83	0.92	0.36	0.44	0.7
Glycine	1.75	0.81	4.19	0.00	4.22	0.0
Crotonic acid (NIST EL)	1.75	0.81	1.71	0.09	1.04	0.4
11-Deoxyprostaglandin E1 (NIST EL)	0.59	-0.75	-0.65	0.52	0.28	0.8
2,6-Dihydroxybenzoic acid	1.64	0.71	2.80	0.00	2.21	0.1
1-Methylhistamine (hydrochloride) (Microbiome)	0.62	-0.69	-0.92	0.35	0.45	0.7
cis-4,7,10,13,16,19-Docosahexaenoic acid (NIST EL)	0.62	-0.69	-0.86	0.39	0.41	0.7
Danazol	0.64	-0.65	-1.17	0.24	0.61	0.6
D-Lysine	0.64	-0.65	-1.09	0.27	0.56	0.6
Deoxycytidine	0.64	-0.64	-0.84	0.40	0.39	0.7
L-Acetylcarnitine	0.64	-0.64	-1.74	0.08	1.07	0.4
cis-4,10,13,16-Docosatetraenoic acid (NIST EL)	0.64	-0.63	0.73	0.47	0.33	0.7
Phenylacetyl L-Glutamine (Microbiome)	0.66	-0.60	-1.61	0.11	0.95	0.4
Butyryl-L-carnitine (chloride) (Microbiome)	0.66	-0.60	0.30	0.76	0.12	0.9
15S-Hydroperoxy-11Z,13E-eicosadienoic acid (NIST EL)	0.67	-0.58	-0.23	0.81	0.09	0.9
E Eleutheroside E +NH3	1.48	0.57	-1.77	0.08	1.09	0.4

Glutathione	1.46	0.54	0.59	0.55	0.26	0.8
L-Phenylalanine	0.69	-0.53	-1.06	0.29	0.53	0.6
(6E)-8-Methyl-6-nonenoic acid (NIST EL)	0.69	-0.53	-2.02	0.04	1.32	0.3
1.alpha.-Methyl-5.alpha.-androstan-3.alpha.-ol-17-one (NIST EL)	1.43	0.52	0.96	0.34	0.47	0.7
Uridine 5-monophosphate	1.42	0.50	2.15	0.03	1.47	0.3
Phosphocreatine	1.41	0.49	2.10	0.03	1.42	0.3
Tetramethylthiourea (NIST EL)	1.40	0.49	2.45	0.01	1.79	0.2
Kaurenoic acid	0.71	-0.49	-0.18	0.86	0.07	0.9
D-Erythronic acid potassium salt (Microbiome)	1.38	0.46	1.72	0.08	1.05	0.4
Didodecyl 3,3'-thiodipropionate (NIST EL)	0.73	-0.46	0.06	0.95	0.02	0.9
Oleic acid	0.73	-0.46	2.17	0.03	1.49	0.3
Arachidonic Acid (sodium salt) (Microbiome)	0.73	-0.46	0.14	0.88	0.05	0.9
Cyclo(-L-Leu-L-Pro) (Microbiome)	0.73	-0.45	-0.96	0.34	0.47	0.7
Taurine	0.73	-0.45	0.06	0.94	0.02	0.9
Glycocholic acid	1.36	0.44	-0.88	0.38	0.42	0.7
Geranyl caprylate (NIST EL)	0.74	-0.43	0.25	0.80	0.10	0.9

2,3-Dihydroxy-3-methylbutyric acid (NIST EL)	0.75	-0.42	0.15	0.88	0.05	0.9
(-)-Carveol (NIST EL)	1.34	0.42	0.70	0.48	0.31	0.7
L-Leucine	0.75	-0.41	0.53	0.59	0.22	0.8
9E,11E-Octadecadienoic acid (NIST EL)	0.76	-0.40	-1.15	0.25	0.60	0.6
(?)-Cotinine (Microbiome)	0.76	-0.39	-0.52	0.60	0.22	0.8
Dibenzylamine (NIST EL)	1.30	0.38	-0.16	0.87	0.06	0.9
1-Hexadecylamine (NIST EL)	1.29	0.37	1.02	0.31	0.51	0.6
Diketene (NIST EL)	0.78	-0.36	-1.96	0.05	1.27	0.3
5-Aminovaleric acid (Microbiome)	1.28	0.36	1.73	0.08	1.06	0.4
FAD	1.28	0.35	1.89	0.06	1.21	0.4
3.alpha.-Galactobiose (NIST EL)	1.25	0.32	0.60	0.54	0.26	0.8
p-Hydroxyphenyllactic acid (NIST EL)	0.80	-0.31	-1.68	0.09	1.02	0.4
Octanoyl-L-carnitine (chloride) (Microbiome)	0.81	-0.30	-0.60	0.55	0.26	0.8
Lithocholic acid, MNH4	1.23	0.30	1.00	0.32	0.49	0.6
D-Glutamine	1.21	0.28	1.45	0.15	0.82	0.5
Decanoyl-L-carnitine (Microbiome)	0.83	-0.27	-1.07	0.29	0.54	0.6

L-Serine	1.20	0.27	3.01	0.00	2.49	0.0
L-Histidine	1.20	0.26	-0.03	0.97	0.01	0.9
Sarcosine	1.19	0.25	0.80	0.42	0.37	0.7
(+)-.alpha.-Tocopherol (NIST EL)	1.19	0.25	0.85	0.40	0.40	0.7
Pyrrolidonecarboxylic acid	1.18	0.23	2.02	0.04	1.33	0.3
Indole-6-carboxaldehyde (NIST EL)	0.85	-0.23	-1.50	0.13	0.86	0.5
L-Alanine	1.17	0.23	1.34	0.18	0.74	0.5
L-Glutamic acid	1.17	0.22	1.80	0.07	1.12	0.4
Nigerose (NIST EL)	1.16	0.22	0.28	0.78	0.11	0.9
??? Cordycepin	0.86	-0.21	-1.28	0.20	0.69	0.5
D-Lactic acid	1.15	0.20	1.01	0.31	0.50	0.6
L-Carnitine	0.88	-0.18	-0.82	0.41	0.38	0.7
Guanidinosuccinic acid	1.13	0.17	1.70	0.09	1.03	0.4
Ethanaminium,2-[[[(2R)-2,3-dihydroxypropoxy]hydroxyphosphinyl]oxy]-N,N,N-trim (Microbiome)	1.12	0.17	1.73	0.08	1.06	0.4
Taurodeoxycholic acid	0.89	-0.17	-0.60	0.54	0.26	0.8
4-Phenylbutyric acid (NIST EL)	1.12	0.17	1.14	0.25	0.59	0.6

Uridine	0.90	-0.16	-1.16	0.25	0.60	0.6
L-Threitol (NIST EL)	1.11	0.15	0.51	0.61	0.21	0.8
1-Methyladenosine	0.90	-0.15	-0.52	0.60	0.22	0.8
Gluconic acid	0.90	-0.15	-0.73	0.46	0.33	0.7
L-Proline	1.10	0.14	0.43	0.66	0.18	0.8
Inosine	0.91	-0.14	0.18	0.86	0.06	0.9
N-Acetyl-Asp-Glu (Microbiome)	1.10	0.14	1.45	0.14	0.83	0.5
5-Aminopentanoic acid	0.91	-0.14	0.41	0.68	0.17	0.8
5-Methoxysalicylic acid	1.09	0.12	1.26	0.20	0.68	0.5
Piperine (Microbiome)	0.92	-0.12	-0.68	0.49	0.30	0.7
Sodium glycochenodeoxycholate (Microbiome)	1.08	0.11	-0.83	0.41	0.39	0.7
1-methyl-6-oxo-pyridine-3-carboxamide (Microbiome)	0.93	-0.11	0.25	0.80	0.09	0.9
3-Oxostearic acid (NIST EL)	1.08	0.11	1.05	0.29	0.53	0.6
Niacinamide	1.07	0.10	0.65	0.51	0.28	0.8
Glycodeoxycholic Acid (Microbiome)	1.06	0.09	-0.11	0.91	0.04	0.9
Histamine	0.94	-0.09	-0.36	0.72	0.14	0.8

Dodecanoic acid	1.05	0.08	0.81	0.42	0.38	0.7
2-Phenylpropionaldehyde (NIST EL)	1.05	0.07	0.42	0.67	0.17	0.8
Cytidine monophosphate	0.96	-0.07	1.55	0.12	0.91	0.4
L-Tryptophan	0.97	-0.05	0.49	0.62	0.21	0.8
Adenine	1.02	0.03	-0.13	0.89	0.05	0.9
(2E,4E)-Hexa-2,4-dienoic acid (NIST EL)	1.02	0.03	0.46	0.64	0.19	0.8
1,3-Dicyclohexylurea (NIST EL)	0.98	-0.03	-0.51	0.61	0.21	0.8
Adenosine	1.02	0.03	1.40	0.16	0.78	0.5
Cumylamine (NIST EL)	1.02	0.02	-0.51	0.61	0.21	0.8
Guanosine	0.99	-0.02	0.73	0.46	0.33	0.7
Serotonin	0.99	-0.02	0.01	0.99	0.00	0.9
Phosphorylcholine	0.99	-0.02	-0.01	0.99	0.00	0.9
Inosinic acid	0.99	-0.02	0.28	0.77	0.11	0.9
2-Methoxymethcathinone (NIST EL)	1.01	0.02	-0.42	0.67	0.17	0.8
Adenosine monophosphate	1.01	0.01	1.08	0.28	0.54	0.6
Dendrobine	1.01	0.01	-0.61	0.54	0.27	0.8

Adenosine 2,3-cyclic phosphate	0.99	-0.01	0.15	0.88	0.05	0.9

FC (Fold Change) – the ratio of metabolite levels in IBS and healthy (IBS/healthy); log_2(FC) – the log_2 transformation of FC; t.stat – t-statistic.

Supplementary Table S2. Fold change analysis and t-test for comparing IBS-C and IBS-D.

Metabolite	Fold Change	log2(FC)	t.stat	p.value	-log10(p)	FDR
Glycodeoxycholic Acid (Microbiome)	0.10	-3.39	-0.42	0.682	0.17	0.92
Sodium glycochenodeoxycholate (Microbiome)	0.20	-2.31	-0.35	0.730	0.14	0.92
E Eleutheroside E +NH3	4.44	2.15	0.71	0.489	0.31	0.92
Glycocholic acid	0.29	-1.77	-0.24	0.814	0.09	0.92
Ursodeoxycholic acid, MNH4	3.18	1.67	1.42	0.176	0.76	0.92
Canrenone	3.01	1.59	0.40	0.692	0.16	0.92
3.alpha.-Galactobiose (NIST EL)	0.34	-1.57	-0.28	0.784	0.11	0.92
Citrulline	0.38	-1.38	-0.35	0.730	0.14	0.92
11-Deoxyprostaglandin E1 (NIST EL)	0.39	-1.36	-2.08	0.052	1.29	0.92
Mannitol	2.54	1.34	0.39	0.700	0.16	0.92
Nigerose (NIST EL)	0.40	-1.33	-0.47	0.646	0.19	0.92
1-Methyladenosine	0.41	-1.30	-1.01	0.328	0.48	0.92
Dodecanoic acid	0.41	-1.30	-0.68	0.506	0.30	0.92
9E,11E-Octadecadienoic acid (NIST EL)	0.42	-1.25	-1.82	0.085	1.07	0.92
Hyocholic Acid (Microbiome)	0.42	-1.24	-0.29	0.777	0.11	0.92
L-Histidine	2.10	1.07	0.88	0.390	0.41	0.92
Phenylacetyl L-Glutamine (Microbiome)	0.48	-1.07	-1.24	0.232	0.63	0.92
1-Hexadecylamine (NIST EL)	0.48	-1.07	-1.42	0.174	0.76	0.92
Crotonic acid (NIST EL)	1.94	0.96	0.13	0.900	0.05	0.94
Didodecyl 3,3'-thiodipropionate (NIST EL)	0.52	-0.96	-0.51	0.616	0.21	0.92

Geranyl caprylate (NIST EL)	0.53	-0.91	-0.47	0.645	0.19	0.92
cis-4,7,10,13,16,19-Docosahexaenoic acid (NIST EL)	1.87	0.90	1.33	0.219	0.66	0.92
L-Acetylcarnitine	1.74	0.80	0.89	0.383	0.42	0.92
Taurocholic acid sodium salt hydrate (Microbiome)	0.59	-0.77	-0.19	0.853	0.07	0.94
3-Hydroxybenzoic acid	0.59	-0.76	-0.54	0.597	0.22	0.92
Sarcosine	0.59	-0.76	-0.82	0.426	0.37	0.92
(+)-.alpha.-Tocopherol (NIST EL)	1.66	0.73	1.17	0.258	0.59	0.92
N-Acetyl-Asp-Glu (Microbiome)	1.61	0.68	1.88	0.081	1.09	0.92
2-Methoxymethcathinone (NIST EL)	0.65	-0.61	-0.83	0.420	0.38	0.92
Taurine	1.52	0.60	1.63	0.135	0.87	0.92
Deoxycytidine	1.51	0.60	0.99	0.335	0.48	0.92
Lithocholic acid, MNH4	0.66	-0.59	-1.71	0.108	0.97	0.92
1.alpha.-Methyl-5.alpha.-androstan-3.alpha.-ol-17-one (NIST EL)	1.50	0.59	0.54	0.596	0.22	0.92
Oleic acid	0.67	-0.58	-0.63	0.540	0.27	0.92
Gluconic acid	1.45	0.54	1.32	0.204	0.69	0.92
4-Phenylbutyric acid (NIST EL)	1.42	0.51	1.69	0.111	0.95	0.92
D-Glutamine	1.42	0.51	1.59	0.132	0.88	0.92
Indole-6-carboxaldehyde (NIST EL)	1.41	0.49	0.37	0.715	0.15	0.92
(?)-Cotinine (Microbiome)	1.40	0.48	-0.91	0.376	0.42	0.92
D-Erythronic acid potassium salt (Microbiome)	0.72	-0.48	-0.30	0.768	0.11	0.92
L-Threitol (NIST EL)	1.36	0.45	1.00	0.341	0.47	0.92
N-Acetylneuraminic acid	0.74	-0.43	-0.30	0.764	0.12	0.92
Adenosine monophosphate	1.34	0.43	1.41	0.177	0.75	0.92
cis-4,10,13,16-Docosatetraenoic acid (NIST EL)	0.74	-0.42	0.45	0.657	0.18	0.92
Cyclo(-L-Leu-L-Pro) (Microbiome)	1.33	0.41	0.23	0.818	0.09	0.92

Adenine	0.75	-0.41	-0.58	0.570	0.24	0.92
Glutathione	0.76	-0.40	0.05	0.959	0.02	0.98
Uridine	0.76	-0.40	-0.90	0.378	0.42	0.92
Linoleic acid	0.76	-0.39	-0.25	0.803	0.10	0.92
2,3-Dihydroxy-3-methylbutyric acid (NIST EL)	1.31	0.39	-0.57	0.576	0.24	0.92
Piperine (Microbiome)	0.77	-0.38	-0.30	0.768	0.11	0.92
Adenosine 2,3-cyclic phosphate	0.78	-0.37	-0.38	0.709	0.15	0.92
5-Methoxysalicylic acid	1.28	0.36	1.51	0.158	0.80	0.92
(-)-Carveol (NIST EL)	0.78	-0.36	-0.72	0.480	0.32	0.92
Guanidinosuccinic acid	1.27	0.35	1.12	0.279	0.55	0.92
Cumylamine (NIST EL)	0.79	-0.33	-0.16	0.877	0.06	0.94
Arachidonic Acid (sodium salt) (Microbiome)	1.25	0.32	0.78	0.447	0.35	0.92
Serotonin	1.25	0.32	-0.75	0.467	0.33	0.92
Inosinic acid	1.25	0.32	0.32	0.756	0.12	0.92
Sphinganine	1.24	0.31	0.41	0.689	0.16	0.92
Diketene (NIST EL)	1.24	0.31	1.19	0.252	0.60	0.92
L-Tryptophan	0.82	-0.29	-0.59	0.560	0.25	0.92
L-Proline	1.22	0.28	0.92	0.371	0.43	0.92
2,6-Dihydroxybenzoic acid	0.82	-0.28	-1.59	0.138	0.86	0.92
Phosphocreatine	0.82	-0.28	-0.61	0.550	0.26	0.92
2-Phenylpropionaldehyde (NIST EL)	1.20	0.26	0.51	0.614	0.21	0.92
1,3-Dicyclohexylurea (NIST EL)	1.19	0.26	0.12	0.904	0.04	0.94
D-Lysine	0.84	-0.25	-1.01	0.328	0.48	0.92
Histamine	1.19	0.25	-0.04	0.967	0.01	0.98
Decanoyl-L-carnitine (Microbiome)	0.84	-0.25	0.63	0.542	0.27	0.92

L-Serine	0.86	-0.22	-0.65	0.525	0.28	0.92
Niacinamide	1.17	0.22	0.73	0.477	0.32	0.92
Butyryl-L-carnitine (chloride) (Microbiome)	1.17	0.22	-0.15	0.879	0.06	0.94
Cordycepin	1.15	0.21	0.39	0.703	0.15	0.92
D-Lactic acid	1.13	0.17	0.63	0.540	0.27	0.92
Danazol	1.13	0.17	-0.62	0.546	0.26	0.92
Tetramethylthiourea (NIST EL)	1.12	0.17	0.37	0.718	0.14	0.92
L-Phenylalanine	1.12	0.16	0.97	0.360	0.44	0.92
Isovaleryl-L-carnitine (chloride) (Microbiome)	1.12	0.16	0.67	0.514	0.29	0.92
1-methyl-6-oxo-pyridine-3-carboxamide (Microbiome)	1.10	0.13	0.39	0.698	0.16	0.92
FAD	1.09	0.13	0.51	0.613	0.21	0.92
1-Palmitoylglycerol (NIST EL)	1.09	0.13	-0.41	0.685	0.16	0.92
3-Oxostearic acid (NIST EL)	0.92	-0.12	-0.49	0.631	0.20	0.92
L-Carnitine	1.08	0.12	0.91	0.382	0.42	0.92
Glycine	1.08	0.11	0.28	0.784	0.11	0.92
Ethanaminium,2-[[[(2R)-2,3-dihydroxypropoxy]hydroxyphosphinyl]oxy]-N,N,N-trim (Microbiome)	1.08	0.11	0.71	0.489	0.31	0.92
10-Gingerol	0.92	-0.11	-0.30	0.771	0.11	0.92
Kaurenoic acid	1.08	0.11	0.77	0.457	0.34	0.92
(2E,4E)-Hexa-2,4-dienoic acid (NIST EL)	0.93	-0.10	-0.19	0.855	0.07	0.94
Octanoyl-L-carnitine (chloride) (Microbiome)	0.94	-0.09	0.31	0.762	0.12	0.92
5-Aminovaleric acid (Microbiome)	0.94	-0.09	-1.10	0.288	0.54	0.92
Taurodeoxycholic acid	0.95	-0.08	1.00	0.334	0.48	0.92
Adenosine	1.05	0.07	-0.03	0.978	0.01	0.98
Phosphorylcholine	0.96	-0.06	-0.12	0.905	0.04	0.94

Dibenzylamine (NIST EL)	1.04	0.05	0.28	0.785	0.11	0.92
p-Hydroxyphenyllactic acid (NIST EL)	1.03	0.04	-0.15	0.884	0.05	0.94
Inosine	0.98	-0.04	-0.29	0.775	0.11	0.92
Guanosine	1.02	0.03	-0.02	0.981	0.01	0.98
Uridine 5-monophosphate	0.98	-0.03	0.44	0.666	0.18	0.92
Pyrrolidonecarboxylic acid	1.02	0.02	0.51	0.623	0.21	0.92
Cytidine monophosphate	0.98	-0.02	-0.48	0.641	0.19	0.92
cis-5,8,11-Eicosatrienoic acid (NIST EL)	0.99	-0.02	0.42	0.681	0.17	0.92
1-Methylhistamine (hydrochloride) (Microbiome)	1.01	0.02	0.99	0.336	0.47	0.92
L-Glutamic acid	0.99	-0.01	0.36	0.723	0.14	0.92
L-Leucine	1.00	0.00	0.25	0.804	0.09	0.92
5-Aminopentanoic acid	1.00	0.00	0.82	0.429	0.37	0.92
L-Alanine	1.00	0.00	0.60	0.559	0.25	0.92

FC (Fold Change) – the ratio of metabolite levels in IBS-D and IBS-C (IBS-D/IBS-C); $\log_2$(FC) – the $\log_2$ transformation of FC; t.stat – t-statistic.
